# Is ICSI Risky?

**DOI:** 10.1155/2013/473289

**Published:** 2013-02-26

**Authors:** May Y. W. Wong, William L. Ledger

**Affiliations:** ^1^School of Women's and Children's Health, Faculty of Medicine, University of New South Wales, Randwick, NSW 2031, Australia; ^2^Royal Hospital for Women, Randwick, NSW 2031, Australia

## Abstract

As assisted reproductive technology (ART) methods become the mainstream of infertility treatment, it has become even more critical to reassess its safety. Following the results of a study published by the Robinson Institute in the New England Journal of Medicine, the risk of ART, especially intracytoplasmic sperm injection (ICSI), has never been so closely scrutinized. This paper traces the origins and development of ICSI, assesses the risks documented in the literature, and finally interprets the implications of the study for couples contemplating therapy. We support the need for continued vigilance towards ICSI and the importance in investigating male-factor infertility as a prequel to its use.

## 1. The ICSI Era

In 1992, the potential of intracytoplasmic sperm injection (ICSI) [1] was realised by the documentation of four ICSI pregnancies in women who had otherwise failed to conceive with other existing ART. Their promising results were hailed as a major breakthrough in recalcitrant male-factor infertility. This initial success has since been repeated at thousands of in vitro fertilization (IVF) programs worldwide, and ICSI has become part of mainstream ART. It now comprises 64% of IVF cases in USA [2].

ICSI involves the in vitro injection of preselected spermatozoa into the cytoplasm of a mature oocyte after ovarian superovulation and oocyte retrieval. ICSI quickly became the favoured technique for cases of male-factor infertility, as it was discovered that the basic semen parameters, such as having a low sperm count or less motile sperm [3], had little impact on its success [4, 5]. This was followed by the achievement of high levels of fertilization in the presence of multiple morphological and dysfunctional sperm defects. The boundaries were further widened with the use of ejaculated sperm, cryopreserved-thawed sperm, and in cases of obstructive and nonobstructive azoospermia; sperm extraction from the testis or epididymis [6]. Today, the indications for ICSI have expanded beyond just male-factor infertility and include multiple failed IVF cycles, mixed-factor infertility, and poor fertilization for unknown reasons, such that it is often used in conjunction with IVF as a “safety-net.” However, these latter “indications” have questionable support based on available evidence [7–10]. This paper explores the findings put forward by an Australian group in a recent paper published in the *New England Journal of Medicine.* Davies et al. [11] conclude that “the risk of birth defects associated with ICSI remained increased after multivariate adjustment, although the possibility of residual confounding cannot be excluded.” This has raised attention from the media and the public alike, especially from women with previous and current pregnancies conceived from ICSI. 

## 2. Potential Risks of ICSI

As the number of children born from ICSI procedures has exponentially increased, greater attention has focused on the safety of the procedure. The vast majority of evidence demonstrates that there is no difference in the rates and types of congenital malformation when comparing ICSI and standard IVF pregnancies [12–15]. This microfertilization technique bypasses multiple steps of the natural fertilization process by introducing apparently intact spermatozoa into the ooplasm. It is therefore important to consider the immediate safety of ICSI, as well as any possible long-term implications.

The potential concerns regarding ICSI offspring relate to four general areas of investigation:

1. transmission of genetic anomalies
2. imprinting disorders
3. congenital malformations
4. developmental abnormalities.

*(1) Transmission of Genetic Anomalies*. A considerable proportion of males requiring ICSI have very low sperm counts, which is associated with a greater risk of carrying chromosomal abnormalities [16, 17]. Spermatozoa selection for ICSI is typically based on motility and morphology attributes, without information about the chromosomal status. Spermatozoa with apparently normal morphology may have DNA fragmentation and could be mistakenly selected to fertilize oocytes during ISCI. The authors have proposed that spermatozoa DNA damage is promutagenic and can give rise to mutations after fertilization, as the oocyte attempts to repair DNA damage prior to the initiation of the first cleavage [18]. This may result in fertilization failure, impaired normal embryo development, reduced implantation or pregnancy rate, and even transference of damaged DNA to the new generation [19, 20]. 

Since pregnancy can be achieved in couples wherein the male partner harbors such abnormalities, the risk that male offspring might later manifest disorders including infertility is very real. In fact, there is limited evidence that paternal sex chromosomal disorders, including microdeletions, are rarely transmitted to male ART offspring [21, 22]. Y chromosome microdeletion, observed in 3%–15% of men with severe oligospermia, may be transmitted to ICSI-conceived male offspring [22]. More boys conceived by ICSI were found to have undescended testes [23] and required urogenital surgery [24]. Hormonally, they were found to have lower serum testosterone levels at 3 months [22], which suggests a subtle impairment of Leydig function that could be inherited from their fathers. While current evidence is inconclusive, the future fertility of ART offspring warrants further research. 

*(2) Imprinting Disorders*. There are suggestions of an increased incidence of epigenetic abnormalities after augmentation with ICSI. The developmental process of genetic imprinting involves the exclusive expression of specific genes from only one parent. However, imprinting disorders occur when this imprint is not set correctly. Gamete micromanipulation may lead to altered gene expression of imprinted genes as crucial epigenetic reprogramming events occur during germ cell development and early embryogenesis [25]. Suboptimal culture conditions are known to affect gene expression as well as fetal development [25, 26]. Some of the molecular defects associated with imprinting disorders include Beckwith-Wiedemann syndrome and Angelman syndrome (1 in 100,000 and 1 in 300,000, resp.). Other researchers have refuted these allegations, stating that the absolute incidence of imprinting disorders is very small [27]. As such, more large prospective, multicentre, and cohort studies are needed to assess the validity of such associations. 

*(3) Congenital Abnormalities*. The relationship between ART and congenital malformations was first reported by Hansen et al. [14] who observed a twofold increased risk of major birth defects in children conceived via these methods [14]. The major defects documented were musculoskeletal, cardiovascular, and urogenital abnormalities, in particular hypospadias [28–31]. ASRM reported a 4.2% risk of major congenital malformations in IVF/ICSI cycles [29, 32]. Previous studies have not demonstrated significant differences between IVF and ICSI [14, 33]. However, suggestions that ICSI may be involved with a higher chance of congenital abnormalities has been further strengthened with the study published by Davies et al. [11]. These findings will be discussed in more detail later.

*(4) Developmental Abnormalities*. Despite the genetic risks and congenital malformations that are reported, several studies found no significant differences in the long-term developmental outcome of ICSI offspring. Basatemur provided reassuring information regarding the physical growth of ICSI children up to the age of 12 years [34]. Another study compared found no significant differences in terms of neurodevelopmental outcome at the age of 5.5 years [35]. It will require further epidemiological surveillance especially for more insidious associations such as the risk of cancers and the offspring's risk of future infertility issues to fully ascertain the long-term impact of ICSI.

## 3. Interpretation of the Current Evidence

Given these conflicting results surrounding birth defects and ICSI, it has been impossible to know which infertility treatments differentially increase the rate of birth defects, or whether there is an underlying association with maternal and paternal factors that necessitate infertility treatment in the first place. The study “Reproductive Technologies and the Risk of Birth Defects,” conducted by the Robinson Institute in South Australia, attempts to answer some of these questions. Davies et al. present data from the largest registry to date, comparing 6163 births from ART, including those form IVF, ICSI, and ovulation induction, with some 308,000 unassisted conceptions (that included terminations and stillbirths) over 16 years. They look at rates of birth defects before the age of 5 years from births from each mode of ART, births from mothers with a history of infertility, and births from mothers with neither a history of infertility nor treatment. In analysis adjusted for confounders (e.g., maternal demographics, co-morbidities such as hypertension, and pregnancy complications such as gestational diabetes), the risk of any birth defect with ART was 8.3% compared with 5.8% in natural pregnancies (unadjusted odds ratio, 1.47; 95% CI, 1.33–1.62). ART was associated with an increased rate of any defect and multiple defects, as well as with cardiovascular, urogenital, and gastrointestinal abnormalities, and cerebral palsy. When ART methods were analyzed independently, there was a 9.9% risk of birth defects using ICSI, compared to 7.2% using IVF. This finding will alleviate the concerns of couples that use IVF, as the risk of defect is not increased beyond their biological risk. Conversely, ICSI was 77% more likely than unassisted pregnancies to have a birth defect, compared to 26% for IVF. What was more striking was that when confounders were taken into consideration, this risk for IVF fell to 7%, whilst with ICSI it remained at 57%. After multivariate adjustment, the association between IVF and the risk of any birth defect was no longer significant, whereas the increased risk of any birth defect associated with ICSI remained significant.

These results can be explained by one of two ways; either the ICSI procedure itself is dangerous or there may be some underlying factors associated with the couples that choose ICSI. This is reinforced as the study also found that pregnancies from mothers that have a history of infertility with natural births were also at an increased risk of conceiving a child with birth defects. So part of the chance of having a child with birth defects after assisted conception results from parental factors related to infertility—but it is not clear what those factors are.

A limitation of the study rests in its use of data from 1986 to 2002. Since then, assisted reproduction has advanced from the methods used in embryo fertilization to the introduction of vitrification which allows a woman to freeze their eggs for more than two decades. So are the results from a generation ago still relevant? In addition to the maternal confounders that the study accounts for, many others remain. Davies et al. do not account for unknown paternal infertility factors (paying only marginal attention to the occupation of the male patient). These may be important contributors to the increased chance of a birth defect after ICSI. Earlier studies have demonstrated that ICSI children carry a higher risk of chromosomal aberrations related to the severity of the male infertility [33]; however, Davies et al. do not disentangle data to determine whether this was the case. This raises the question of whether the residual risk that remained was related to parental factors, rather than to the ICSI procedure itself. The reported adverse trends in male reproductive health [36] and the possibility of genetic and environmental effects as determinants of male infertility [37] provide even more reasons to thoroughly address these questions. ICSI may be facilitating the transfer of genetic disorders to future generations by bypassing all natural hurdles for sperm selection without imposing more pertinent selection criteria. At present, the sperm chromatin structure assay (SCSA) is the most commonly used assay for measuring DNA fragmentation with clear and clinically useful cutoff levels for inferring male fertility potential [38–41]. SCSA is a standardized test which measures the percentage of sperm with a high susceptibility to DNA denaturation and is expressed as a DNA fragmentation index (%DFI) [42, 43]. Generally, levels greater than 30% DFI indicate a significantly lower chance of achieving an ongoing pregnancy. Newer methods include the terminal deoxynucleotidyl-transferasemediated dUTP nick-end labelling assay (TUNEL) [41, 44–46] and Comet assay [47] which measure both single- and double-strand DNA breaks in individual cells [48, 49] but have not been fully integrated into clinical practice. Methods such as SCSA and those under development such as TUNEL and Comet assays need to be more fully integrated into routine clinical application. 

There are several explanations for why the results of this study record high risks of birth defects in comparison to similar studies in the past. For their overall results, the authors did not separate twin pregnancies from singleton pregnancies. Twin pregnancies are known to be at higher risk of all birth defects, especially cerebral palsy. They also did not distinguish single embryo transfer from double embryo transfer. Even if it results in a singleton birth, previous research has shown double embryo transfer to be associated with a higher risk of birth abnormalities than single embryo transfer [50]. In addition, the study's definitions used are questionable as, unlike other studies, cerebral palsy was included as a “birth defect.” Many would argue that the cause of cerebral palsy is multifactorial and could be attributable to low gestational age and low birthweight. The conflicting criteria for the definition of birth defects have been described previously; when birth defects reported from Belgium were reassessed based on Western Australian criteria, a twofold higher risk of major birth defects in ICSI children was observed [51]. This highlights the need to standardize definitions in order for results to be compared meaningfully across studies. 

The authors of this study do not discuss the results of a recent Swedish population-based study on 15 570 ART infants (9372 ICSI pregnancies) [52] which demonstrated that the adjusted odds ratio of birth defects for the ICSI versus IVF cohort was 0.90 (95% CI 0.78–1.04). Thus there did not seem to be any apparent risk difference associated with the IVF method. Moreover, the risk of hypospadiasis, which had previously been associated with ICSI, was no longer significantly different from the risk in the general population. This is in accordance with a recent meta-analysis in which the outcomes of 46 studies covering 125,000 IVF and IVF/ICSI babies demonstrated a small increased risk of congenital abnormalities with IVF and ICSI babies compared to natural conception, but no difference in the risk between IVF and ICSI conceptions (RR = 1.05, 95% CI 0.91–1.20) [53]. 

This study reminds us that the success of ICSI has been a double-edged sword. Inarguably, it has gone where IVF has not, offering men who were previously rendered infertile the possibility of becoming fathers. However, simultaneously it has hampered progress on the development/refinement of diagnostic tools (particularly related to spermatozoa-oocyte interaction), and perhaps of medical treatments for male infertility. Ironically, in an age of burgeoning knowledge surrounding reproductive function, the focus on the causes of infertility or subfertility in a couple has decreased. Unfortunately and frequently, patients are enrolled directly into ICSI programs with little clinical and diagnostic workups to avoid the costs of male-infertility testing. Such views have been labeled as “insidiously lazy” [54] and “a dangerous loss of control over the clinical decision-making process” [55]. This poses a risk of nondiagnosis of subjacent etiologic causes, and, more importantly, the presence of hidden diseases, like testicular cancer [37]. Managing male infertility requires more clinical andrology, not less. Instead, many couples are diagnosed with so called “unexplained infertility,” and most men never receive an explanation for the causes lying behind their reduced semen quality. ICSI is (mis)used nowadays almost as insurance for good fertilization employed even when there is no clear evidence of sperm issues that would necessitate its use. In addition, the financial cost of ICSI at an additional $1500–$2000 or 11% more than the costs of IVF [56–58] per cycle may be an impetus for fertility clinics to expand its use. However, costs per ongoing pregnancy of ICSI were lower compared with IVF due to fewer incomplete treatment cycles and higher success rates per cycle. Despite the immediate success of ICSI, there lacks an internal review board and ethics committee to assess its biological impact as well as regulatory action by the Food and Drug Administration. ICSI was offered to thousands of patients without extensive animal research to antedate its use in humans.

## 4. Conclusion

This study shows that it is still not known whether the risks associated with ICSI are related to the procedure or to inherent sperm abnormalities. What is known is that couples with infertility have modest excess risk for having children with birth defects, despite controlling for maternal factors, regardless of how or whether the infertility is managed. More importantly, it highlights the need for continued vigilance, especially with ICSI. There should be more vigorous evaluation and treatment of the male patient as part of the routine evaluation of the infertile couple and not the use of empirical therapy. If ICSI is deemed the most suitable method in the therapeutic armamentarium, then the associated risks of birth defects must be conveyed to the couple. It is imperative that the children being born through ICSI and other ART continue to be monitored. Through these methods, we will hopefully be able to balance improvements in conception rates and at the same time minimize the chance of successful pregnancies resulting in children with birth defects. 
